# Resistance: what is new and on the horizon, and a time to teach old dogs new tricks?

**DOI:** 10.7448/IAS.17.4.19517

**Published:** 2014-11-02

**Authors:** Jonathan Schapiro

**Affiliations:** HIV/AIDS clinic, National Haemophilia Center, Tel Aviv, Israel

## Abstract

Understanding HIV drug resistance has played a key role in the success of antiretroviral therapy. This knowledge allowed for the prediction of resistance evolution when a specific drug or combinations of drugs were administered, informing strategies implemented for initial and subsequent drug regimens. Resistance testing of individual patients detects transmitted as well as acquired drug resistance as a result of treatment failure, leading to improved treatment choices in drug naïve and drug experienced patients accordingly. The last two decades has seen a great deal of evolution, improvement and change in the care of HIV-positive individuals. Much of this was aimed at preventing, reducing or minimizing the impact of HIV drug resistance. Drugs with much improved resistance characteristics were designed and gained widespread use, as well as those with superior pharmacology and toxicity profiles assisting in better adherence and far reduced failure rates. Patient management strategies and monitoring technologies were refined, and education of clinicians and patients on optimal aspects of care routinely was implemented. All these have led to a new world of HIV clinical care and require a rethinking of how best to use our knowledge of resistance and when, how and in whom to test for it. What lays ahead for HIV drug resistance in the near and distant future, and is it time to teach old dogs new tricks?

